# Protocol for the development of a Core Outcome Set for trials on the prevention and treatment of Orthodontically induced enamel White Spot Lesions (COS-OWSL)

**DOI:** 10.1186/s13063-021-05371-w

**Published:** 2021-07-31

**Authors:** Danchen Qin, Yunlei Wang, Colin Levey, Peter Ngan, Hong He, Fang Hua, on behalf of the Core Outcome Set for trials on the prevention and treatment of enamel White Spot Lesions (COS-WSL) group

**Affiliations:** 1 Hubei-MOST KLOS & KLOBM, School & Hospital of Stomatology, Wuhan University, Wuhan, China; 2 Department of Orthodontics, School & Hospital of Stomatology, Wuhan University, Luoyu Rd. 237, Wuhan, 430079 China; 3 School of Dentistry, University of Dundee, Dundee, UK; 4 Department of Orthodontics, West Virginia University, Morgantown, USA; 5 Centre for Evidence-Based Stomatology, School & Hospital of Stomatology, Wuhan University, Luoyu Rd. 237, Wuhan, 430079 China; 6 Division of Dentistry, School of Medical Sciences, Faculty of Biology, Medicine and Health, University of Manchester, Manchester Academic Health Science Centre, Manchester, UK

**Keywords:** White spot lesion, Orthodontics, Core outcome set, Clinical trials

## Abstract

**Background:**

Enamel white spot lesions (WSLs), characterized by an opaque, matt, and chalky white appearance of enamel, are a sign of incipient caries. WSLs are common in orthodontic practice and can affect both the oral health and dental aesthetics of patients. Extensive studies have been conducted to evaluate the effectiveness of prevention or treatment for orthodontically induced enamel WSLs. However, substantial heterogeneity has been found in the outcomes used for the prevention and treatment of WSLs in literature, which prevents researchers from comparing and combining the results of different studies to draw more decisive conclusions. Therefore, we aim to develop a Core Outcome Set for trials on the prevention and treatment of Orthodontically induced enamel White Spot Lesions (COS-OWSL).

**Methods:**

The development of COS-OWSL comprises four phases: (1) a scoping review to identify and summarize all existing outcomes that have been used in trials on the prevention or treatment of orthodontically induced WSLs; (2) qualitative interviews with orthodontic patients without (for prevention) and with WSL-affected teeth (for treatment) and relevant dental professionals to identify additional outcomes relevant to them; (3) Delphi surveys to collect opinions from key stakeholders including patients, dental professionals, and researchers and to reach a preliminary consensus; and (4) a consensus meeting to develop the final COS-OWSL.

**Discussion:**

The COS-OWSL will be developed to facilitate the synthesis of evidence regarding the prevention and treatment of orthodontically induced WSLs and to promote the consistent use of relevant patient-important outcomes among future studies in this field.

**Trial registration:**

Core Outcome Measures in Effectiveness Trials (COMET) initiative (the COS-WSL project) 1399

## Background

Dental caries is one of the most common oral diseases and a significant cause of tooth loss [1]. Although preventable, dental caries still shows high prevalence, and untreated dental caries poses a significant cost burden on oral health services [2]. White spot lesions (WSLs) are areas of demineralized enamel characterized by an opaque, matt, and chalky white appearance without cavitation [3, 4]. As a sign of incipient caries, WSLs can develop into cavitated carious lesions if not treated timely [5].

Poor oral hygiene is considered a major contributor to WSLs [6, 7]. Orthodontic treatment is also an important risk factor, and the incidence of WSLs is significantly higher among orthodontic patients [8]. The appliances used in orthodontic treatment such as brackets, bands, and wires increase the number of plaque retention sites, which add difficulties to the maintenance of good oral hygiene [6, 9]. A recent review showed that the incidence of WSLs in patients who received over 12-month fixed orthodontic treatment was 23–76% [10]. In addition, anterior teeth and premolars develop WSLs more frequently, which can have a negative impact on dental aesthetics [11]. Besides aesthetic concerns, the demineralization can reduce the hardness of enamel and leave it vulnerable to damage, therefore proceeding into cavitation and reducing the lifetime prognosis of the tooth [12].

Despite the high prevalence of orthodontically induced WSLs, the methods used for detecting WSLs varied in previous research [13]. Many tools can be used to detect WSLs, including traditional visual inspection, photograph and radiograph, transillumination, and fluorescence methods [14]. For example, direct visual inspection, the simplest and most cost-effective detection method of WSLs, has been frequently adopted in routine practice, but the specific approach and criteria used in visual inspection are rarely consistent across different investigators and clinical settings [13, 15]. Quantitative light-induced fluorescence evaluation is much more sensitive than direct visual inspection and can detect enamel demineralization before clinically visible [6], but its relevance to patients may be limited.

The diversity of outcomes and outcome measures was another issue in studies evaluating the effectiveness of prevention or treatment of WSLs [16]. Some studies focused on the incidence or prevalence of WSLs to evaluate the treatment effects [7, 11], while others utilized the severity or activity of lesions [17]. Outcomes that researchers used to assess the severity of WSLs include colors [18], sizes or dimensions [19, 20], and surface roughness [21] of lesions. In addition, investigators have employed diverse outcome measures to assess the same outcome [22]. For instance, more than ten scoring criteria for visual inspection were used to classify WSLs [13]. In a previous systematic review, four included studies that performed a direct visual examination for WSLs utilized four different evaluation index systems [23]. The lack of consistency among relevant assessment criteria compromises the comparability of outcomes from clinical trials and evidence synthesis.

Due to the aforementioned inconsistency in WSL evaluation, researchers often fail to make effective comparisons between results from clinical trials [24]. Many systematic reviews about WSLs found it impossible to pool the results due to substantial heterogeneity [17, 25–29]. This prevents the improvement of health care in this area and might pose the risk of continuing ineffective treatments to patients [24].

Extensive evidence has suggested that results from trials and systematic reviews are often irrelevant to patients and health service users. According to Fleming et al. [30], only 22% of outcomes used in dental trials were both patient and clinician centered. The use of patient-reported outcomes may help dental professionals better understand patient’s treatment need and improve oral health care [31, 32]. However, a Cochrane systematic review on fluorides for preventing WSLs during fixed orthodontic treatment found that patient-reported outcome was not included in any of the included trials [33]. Quantitative measurement for WSLs such as optical and fluorescent methods is accurate and useful in research, but it might not be cost-effective and meaningful to patients. Therefore, future research needs to increase the relevance of results to both patients and dental professionals.

To address issues outlined above, it is essential to develop a core outcome set (COS) for trials on the prevention and treatment of orthodontically induced enamel WSLs. Core outcome sets are agreed standardized sets of outcomes that should be measured and reported in all clinical trials for a specific healthcare area [34]. Hence, outcomes in a COS will always be included and reported as a minimum, while researchers can explore and measure other outcomes of interest as well. If an outcome is not included in a particular trial, researchers should determine a priori and explain the reason [35].

At present, a COS for Outcomes in Trials for Management of Caries Lesions (OuTMaC), designed for all types of dental caries, is under development [36]. Although orthodontically induced WSL is one type of dental carious lesion, it tends to affect a narrower population and has different levels of impact on aesthetics and quality of life. In addition, due to its relatively high prevalence in the anterior teeth, it may result in different outcomes of interest and affect the final COS. The scope of OuTMaC is restricted to the management of existing carious lesions and does not provide a COS for the prevention of dental caries. Therefore, the development of COS-OWSL will facilitate outcome reporting and evidence synthesis in trials on the prevention or treatment of orthodontically induced WSLs.

Tsichlaki et al. [37] recently published a COS for orthodontic treatment which included WSLs in its “adverse effects and/or events” domain. However, with “malocclusion” being its health condition and “orthodontic treatment” being its intervention of interest, the orthodontic COS was essentially developed for trials evaluating the effectiveness of orthodontic therapies, and therefore not adequate and applicable for trials on the prevention and treatment of orthodontically induced WSLs. Therefore, to address the existing outcome heterogeneity and facilitate future evidence synthesis in this area, a specifically developed COS is needed. In addition, as the COS for orthodontic treatment has determined *what* should be measured [37], the next stage for our profession is to determine *how* and *when* each outcome should be measured. In this aspect, the development of a COS for WSLs can provide support to future efforts regarding how “adverse effects and/or events” should be measured.

### Objectives

This article aims to present a protocol for the development of a COS for trials on the prevention or treatment of Orthodontically induced enamel White Spot Lesions (COS-OWSL). In this study, we will develop a consensus about “what” to measure for orthodontically induced WSLs. “How” and “when” to measure will be determined in future research. The objectives of this study are:

1. To identify all existing outcomes that have been used in trials on the prevention or treatment of orthodontically induced enamel WSLs by a scoping review
2. To identify additional outcomes relevant to patients and dental professionals by qualitative interviews and to develop a list of candidate outcomes
3. To collect opinions from key stakeholders including patients, dental professionals, and researchers and to reach a preliminary agreement through online Delphi surveys
4. To achieve a final consensus on COS-OWSL by a consensus meeting

### Scope

The scope of this COS-OWSL is:

- Health condition: orthodontically induced enamel WSLs in primary or permanent teeth
- Target population: orthodontic patients without (for prevention) and with orthodontically induced WSL-affected teeth (for treatment), without any age, sex, or dentition restrictions
- Intervention: all interventions to prevent or treat orthodontically induced WSLs
- Settings: clinical trials, other types of clinical research, and dental clinical practice

## Methods

The COS-WSL project was registered on the Core Outcome Measures in Effectiveness Trials initiative (COMET) website (http://www.comet-initiative.org/studies/details/1399) which includes ongoing and completed COS protocols and studies in various medical fields, all of which are freely available to the public. The present study protocol was written in accordance with the Core Outcomes Set-STAndardised Protocol Items (COS-STAP) [38] and the Core Outcome Measures in Effectiveness Trials (COMET) Handbook [39].

The COS-OWSL will be developed through a four-phase process: a scoping review for existing outcomes, qualitative interviews, a two-round Delphi survey, and a consensus meeting. Patients will be involved in the qualitative interviews, Delphi survey, and consensus meeting to provide their preferences and choice of outcomes regarding orthodontically induced WSLs.

### Phase 1: Scoping review

To determine what to measure and to identify the existing knowledge, we will conduct a scoping review of all outcomes used in published clinical trials regarding the prevention and treatment of orthodontically induced WSLs and develop an inclusive list of the outcomes. The methods and findings of this scoping review will be reported in accordance with the PRISMA extension for Scoping Reviews (PRISMA-ScR) [40].

In this review, we will search three databases: MEDLINE (via PubMed), Embase, and the Cochrane Central Register of Controlled Trials (CENTRAL). The dates of coverage will be restricted to the most recent 10 years to obtain an up-to-date pool of relevant outcomes [39]. We will use both free-text and subject headings in our search strategy, based on the following main concepts: “tooth demineralization” OR “white spot lesion,” “orthodontics,” and “randomized controlled trial” OR “controlled clinical trial.” In addition to electronic searches, we will hand search references cited in those identified eligible studies and relevant systematic reviews. A search of grey literature will not be performed in this scoping review, as it is considered unlikely to provide new relevant outcomes or significantly alter the final list of the outcomes [41].

The inclusion criteria will be as follows: (1) study type, randomized controlled trials (RCTs) and controlled clinical trials (CCTs); (2) target population, orthodontic patients without (for prevention) and with orthodontically induced WSL-affected teeth (for treatment) with any dentition and in any health condition from both genders and all age groups; (3) intervention, an intervention for preventing or treating WSLs should be implemented and compared with another intervention or a placebo; and (4) outcome, a minimum of one subjective or objective outcome measure should be clearly reported. No restrictions on settings or follow-up periods will be adopted. The exclusion criteria will be in vitro or in situ studies, case reports, case series, observational studies, studies reported in languages other than English, and publications without available full texts.

Two authors will screen the identified records independently and in duplicate, according to the abovementioned eligibility criteria. Titles and abstracts will be screened first, and if they lack sufficient information to make a judgment, the corresponding full texts will be obtained and examined. From each included study, two authors will extract verbatim the study characteristics and outcomes used [39]. A pilot study will be conducted to calibrate the authors and develop a template for data extraction using 10% of all included studies (randomly selected). Thereafter, two authors will extract data from the rest of included studies independently and in duplicate. Any discrepancy during screening and data extraction will be settled through discussion with the other authors.

After data extraction, outcomes with similar definitions but different terminology will be merged into a single standard outcome. The outcomes of WSL prevention and treatment will be listed separately. Then, two authors will categorize these outcomes into outcome domains. Any discrepancy in the outcome classification will be discussed through group consensus among all authors. Finally, a preliminary list of outcome domains will be developed accordingly to provide information for the following phases.

### Phase 2: Qualitative interviews

After the scoping review for all the existing outcomes for orthodontically induced WSLs, qualitative interviews will be performed to identify and fill the evidence gaps and to determine outcomes relevant to patients and dental professionals. The purpose of the qualitative interviews is to understand the perspectives of patients and dental professionals on orthodontically induced WSLs, and to ensure that no important outcomes are overlooked [39, 42].

Purposeful sampling will be used to recruit information-rich participants for the qualitative interviews to include as diverse ranges of opinions from patients and dental professionals as possible [43]. The recruitment of patients and dental professionals will be conducted in China. We will recruit patients with different ages, genders, occupations, types of WSL-affected teeth (primary or permanent, anterior or posterior), severity of WSLs, and experience of any intervention for WSLs (prevention or treatment). Patients under 18 years old will be allowed to express their opinions, and opinions from their parents or carers who are familiar with their condition will also be collected to form combined opinions [44]. We will also recruit dental professionals with different ages, genders, dental specialties, and level of experience in the prevention or treatment of WSLs.

Semi-structured interview questions will be developed to help ask all participants similar questions and will be piloted with a small group of patients and dental professionals. A dentist and researcher will interview the patients and dental professionals through face-to-face interviews or phone calls one by one. The researcher will be familiarized with the questions and receive training in interview skills prior to interviews. Data collection from interviews will be ended until data saturation is reached, which is defined as no generation of new themes and outcomes. When no new themes or outcomes emerge, we will continue to interview two more participants to confirm the saturation of data [45, 46].

The content of the interviews will be transcribed and analyzed. To protect respondents’ privacy, the interview will be anonymized, and all the raw data will be stored in one author’s computer and kept confidential. A content analysis will be conducted to identify outcomes or outcome domains reported by patients and dental professionals. Two authors will perform the coding procedure for the content analysis independently and in duplicate and then categorize the responses into the predefined outcome domains, with any disagreement resolved through discussion with the other authors. Only new outcomes or outcome domains that are suggested by two or more participants will be included in the outcome list [47].

Two authors will combine the outcomes from the qualitative interviews with the outcomes identified from the scoping review and develop a list of outcomes relevant to researchers, patients, and dental professionals for the following consensus process.

### Phase 3: Delphi survey

The online Delphi survey will consist of two-round anonymous questionnaires to specify which outcomes the key stakeholders consider most important and to reach a preliminary consensus among them.

Purposeful sampling strategies will be adopted to recruit a diverse, international participant pool for the Delphi survey, with involvement from three stakeholder groups: patients, dental professionals, and researchers [48]. We will send potential participants a personalized email to explain the objectives of the survey and invite them to participate. To minimize attrition, the importance of their completion of the whole 2-round Delphi process will be emphasized, and individuals who agree to complete both rounds of the survey will be included. Before the survey, we will send emails to ask participants to opt in. Then, we will provide a link to the Delphi survey and the contact information of investigators to the participants who agree to participate by email. Participants who do not complete the questionnaire in *round 1* will not be invited for *round 2*. In both rounds of the survey, email reminders will be sent every 2 weeks to participants who have not finished the survey to maximize completion rate. Each round of the survey will last for a month; if the response rate of *round 2* is low, we will keep the survey open for another 2 weeks and send additional reminders to minimize potential attrition bias [39].

Both medical terms and plain language descriptions will be provided in the questionnaire to avoid ambiguity [48]. The outcomes will be voted for prevention and treatment separately and presented by outcome domains. Outcome domains and outcomes within each domain will be presented in a random order. Prior to the official release, the full text of the questionnaire will be piloted with several representatives from each stakeholder group to refine the language and increase readability for all stakeholders.

In *round 1* of the Delphi survey, participants will be provided with the list of outcome domains with candidate outcomes to decide which outcomes are most important. The importance of each candidate outcome will be rated on a 1 to 9 scale recommended by the GRADE group, in which 1 to 3 represents that an outcome is of limited importance, 4 to 6 important but not critical, and 7 to 9 critical [49]. At the end of the *round 1* survey, the participants will also be asked to list outcomes that they consider relevant but not included in the questionnaire. For the existing outcomes, the participants will also be allowed to make suggestions to improve the clarity of the wording. We will revise the relevant wording of the terms and definitions accordingly for the subsequent round.

The responses from *round 1* will be summarized by calculating the percentage of participants scoring each score from 1 to 9 by stakeholder groups. Suggestions for new outcomes in the first round Delphi survey will be reviewed by two researchers independently and in duplicate. For responses that are unclear or ambiguous, we will ask the respondents for clarification. Only new outcomes suggested by two or more participants will be included. After removing duplicate responses and responses that show up only once, all remaining responses will be grouped into domains. If any new outcomes cannot be grouped into the existing domains, new outcome domains will be developed accordingly.

A greater number of outcomes can significantly decrease the response rates in a Delphi survey [50]. Therefore, to reduce attrition and the burden on respondents, only outcomes that meet the following criteria will be included in the second round of the survey: more than 50% of the respondents score between 7 and 9 and less than 15% score between 1 and 3 [51]. Additional outcomes suggested by at least 2 participants in *round 1* will also be rated in *round 2*. On the top of the *round 2* questionnaire, the summary results of *round 1* will be fed back to each participant along with their own scoring in *round 1*. At the end of the *round 2* questionnaire, the participants will be asked to rank their top 10 choices in order of importance [52].

The participants will be divided into two groups—patients and experts (dental professionals and researchers)—to summarize the rating scores and to evaluate consensus. The consensus criteria will be consistent with a previous study on COS development [51]. Consensus is divided into *consensus in* and *consensus out*. *Consensus in*, which means the outcome should be included in the COS, will be defined as at least 70% of participants in each group scoring between 7 and 9 and less than 15% scoring between 1 and 3 for an individual outcome. *Consensus out*, which means the outcome should be excluded from the COS, will be defined as at least 70% of participants in each group scoring between 1 and 3 and less than 15% scoring between 7 and 9. All other score distributions will be considered as a lack of consensus for the inclusion in the COS. Outcomes that remain undetermined after the 2-round Delphi survey will be discussed in a consensus meeting for the final COS-OWSL. In addition, we will analyze how individual scores change through the Delphi process to show if participants are moving towards consensus or not.

### Phase 4: Consensus meeting

To achieve a final consensus on COS-OWSL, an online international consensus meeting will be held after the Delphi survey. A group of key stakeholders will be invited to the meeting to discuss the remaining outcomes that have not reached a consensus in the Delphi survey, the definition and terminology of included outcomes, and the number of outcomes to be included in the final COS-OWSL. Outcomes that achieve consensus and are included in the COS-OWSL (consensus in) after the two-round survey will also be discussed and voted to confirm their importance.

The key stakeholders attending the consensus meeting will include representatives of patients, dental professionals, and researchers. Attendees will be selected broadly from those who have extensive knowledge or experience of orthodontically induced WSLs. From each group of stakeholders, we will also invite all participants who have completed both rounds of the Delphi survey to attend the consensus meeting.

A researcher who does not engage in discussion or voting will host the online consensus meeting. The results of the Delphi survey will be presented to all attendees at the consensus meeting. The outcomes that have not reached consensus in the Delphi survey will be discussed in detail. The attendees will be asked to offer their perspectives on the importance of each outcome. Then, they will vote for each outcome as “in” or “out” for the inclusion in the final COS-OWSL. Voting will be conducted anonymously using an online application. A researcher not involved in the meeting will collect and analyze all data. Feedback will be provided to the attendees in descriptive statistics after each round of voting. Consensus will be reached on an outcome when the majority (>70%) vote as “in” or “out”, and no further voting of that outcome will be required. Outcomes that do not achieve a consensus will be further discussed to explore and resolve divergence. The meeting will alternate between voting and discussion for three rounds. If no consensus is achieved in an outcome for three rounds of discussion and voting, the outcome will not be further voted and included in the final COS-OWSL. Following the above process, we will develop the final COS-OWSL and present the outcomes by appropriate outcome domains.

### Dissemination

The study results will be disseminated through peer-reviewed academic journals and international conferences. To promote the uptake of the COS-OWSL, we will engage with relevant groups/organizations such as the Cochrane Oral Health Group and International Association for Dental Research. We will also provide all the participants with a summary of the results.

## Discussion

The COS-OWSL study will develop a COS for use in clinical trials regarding the prevention or treatment of orthodontically induced WSLs. The development of COS-OWSL will facilitate and justify the selection of outcomes and standardize the reporting of results for future research. Therefore, the COS-OWSL will help minimize outcome heterogeneity and reporting bias and thereby facilitate data syntheses and help reduce avoidable research waste [53, 54].

Outcomes used in clinical trials have been chosen largely by the trialists or academics and are rarely patient or clinician centered. To identify patient- and dentist-relevant outcomes, qualitative interviews with patients and dental professionals will be performed in this study to ensure no important outcomes are missed. In addition, we will involve a wide range of key stakeholders including patients, dental professionals, and researchers in the development of COS-OWSL to collect different and representative opinions, and thereby develop a commonly accepted COS-OWSL. The final COS-OWSL will be of adequate clinical relevance and will be able to help improve dental clinical practice and patient care.

## Study status

The scoping review to identify and summarize the existing outcomes has been completed.

## Data Availability

Not applicable.

## Abbreviations

CCT: Controlled clinical trial
COMET: Core Outcome Measures in Effectiveness Trials
COS: Core outcome set
OWSL: Orthodontically induced enamel White Spot Lesions
RCT: Randomized controlled trial
WSLs: White spot lesions
